# Role of *Lactobacillus pentosus* Strain b240 and the Toll-Like Receptor 2 Axis in Peyer's Patch Dendritic Cell-Mediated Immunoglobulin A Enhancement

**DOI:** 10.1371/journal.pone.0091857

**Published:** 2014-03-14

**Authors:** Yoshifumi Kotani, Jun Kunisawa, Yuji Suzuki, Ikutaro Sato, Takao Saito, Masamichi Toba, Noriyuki Kohda, Hiroshi Kiyono

**Affiliations:** 1 Otsu Nutraceuticals Research Institute, Otsuka Pharmaceutical Co., Ltd., Shiga, Japan; 2 Division of Mucosal Immunology, Department of Microbiology and Immunology, The Institute of Medical Science, The University of Tokyo, Tokyo, Japan; 3 International Research and Development Center for Mucosal Vaccines, The Institute of Medical Science, The University of Tokyo, Tokyo, Japan; 4 Department of Medical Genome Science, Graduate School of Frontier Science, The University of Tokyo, Chiba, Japan; 5 Graduate School of Medicine, The University of Tokyo, Tokyo, Japan; 6 Core Research for Evolutional Science and Technology (CREST), Japan Science and Technology Agency, Tokyo, Japan; 7 Laboratory of Vaccine Materials, National Institute of Biomedical Innovation, Osaka, Japan; Institut National de la Santé et de la Recherche Médicale U 872, France

## Abstract

Lactic acid bacteria are well known to possess immune-modulating effects, but the mechanisms underlying their modulation of the gut immune system are not fully understood. Here, we examined the localization of heat-killed *Lactobacillus pentosus* strain b240 (b240) in intestinal tissues and the effect of b240 on adaptive immune cascades in the gut. Histological analysis showed that b240 co-localized with dendritic cells (DCs) in the subepithelial dome region of Peyer's patches (PPs). In a PP cell culture system, b240 promoted the production of immunoglobulin A (IgA), interleukin (IL)-6, IL-10, interferon (IFN)-γ, and tumor necrosis factor, but not IL-4, IL-5, B-cell activating factors, IFN-α, IFN-β, and transforming growth factor-β1. The enhanced IgA production by b240 was attenuated by neutralizing IL-6, a potent IgA-enhancing cytokine. b240 stimulated DCs to produce an elevated amount of IL-6 in a Toll-like receptor (TLR) 2-, but not TLR4- or TLR9-dependent manner. Finally, we demonstrated that TLR2-mediated IL-6 production from PP DCs in response to b240 activated B cells to produce a large amount of IgA in a DC-B cell co-culture system. Our findings open up the possibility that the heat-killed form of *Lactobacillus pentosus* strain b240 can be used as a TLR2-mediated DC-activating biologic for enhancing IgA production in the intestine.

## Introduction

Gut mucosal epithelial surfaces are in continuous contact with a heterogeneous population of endogenous microbiota and are exposed to foods, exotic microbes, and viruses [1], [2]. The gut thus establishes unique surveillance and defensive mechanisms as well as a symbiotic immune system [3], [4]. One of unique features of the intestinal immune system is a highly specialized antibody inclination towards immunoglobulin A (IgA) production. The secretory form of IgA (SIgA) antibodies has been shown to play critical roles in both the protective and symbiotic phases of intestinal immunity. SIgA thus prevents the invasion of pathogens by inhibiting their binding to intestinal epithelial cells and neutralizing their derived toxins [6]–[8]. At same time, SIgA maintains the appropriate composition of commensal bacteria [6]–[8]. For the production of SIgA, gut-associated lymphoid tissues (GALT) such as Peyer's patches (PPs) are an important inductive site for the initiation and generation of antigen (Ag)-specific IgA-committed B cells [5]. In PPs, Ag-specific CD4^+^ T cells are primed and activated by dendritic cells (DCs) to support IgA class switch recombination (CSR) of IgM-positive B cells to IgA-positive B cells by using transforming growth factor (TGF)-β, interleukin (IL)-4, and CD40 ligand [34]. Recently, several studies revealed that PP DCs induce IgA CSR via a T cell-independent pathway by producing retinoic acid (RA) [23] or a proliferation-inducing ligand (APRIL) and B-cell activating factor (BAFF) [24], [25]

*Lactobacillus* species are commensal bacteria in the human gastrointestinal tract and are widely used in manufacturing fermented food products [9]. Certain *Lactobacillus* strains are classified as probiotics, and are proposed as live microbial food ingredients or components of microbial cells that are beneficial to health [41], [42]. There is increasing evidence that specific probiotic *Lactobacillus* strains influence host innate and adaptive immune responses, such as the pro-inflammatory/anti-inflammatory responses of antigen-presenting cells, T cell differentiation, and antibody production [9], [10], [26]. Clancy proposed the term “immunobiotics” in probiotics to identify bacteria that promote health by driving mucosal immune mechanisms, compared to those with strictly local effects such as the alteration of microbiological balance [32].

In our previous studies, we showed that the heat-killed *Lactobacillus pentosus* strain b240 (b240) had the immunological effect of enhancing IgA production in PP cell culture among the 150 lactic acid bacteria tested, and that the oral administration of heat-killed b240 in mice promoted IgA production [13], [14]. Furthermore, oral intake of drinking water supplemented with heat-killed b240 accelerated salivary IgA secretion in healthy adults and the elderly [15], [16].

It has been shown that specific *Lactobacillus* strains promote IgA production or increase the number of IgA^+^ B cells in the small intestines of mice [11], [12], [27]–[29]. It was reported that isolated PP cells exposed to *Lactobacillus* GG *in vivo* showed elevated secretion of IgA accompanied by increased IL-6 production in culture [27]. The other study showed that the oral administration of *Lactobacillus paracasei* subsp. *paracasei* NTU 101 increased the number of IgA^+^ B cells in the small intestinal lamina propria with strong DC-CD4^+^ T cell interaction through an increased frequency of CD40 ligand^+^CD4^+^ T cells [28]. It was also shown that the oral administration of milk fermented by *Lactobacillus casei* DN114001 increased the number of IgA^+^ B cells in the small intestine with increasing IL-6 secretion from intestinal epithelial cell culture *ex vivo* [29]. All of these previous studies provided supporting evidence for the immunobiotic nature of *Lactobacillus* species. However, the precise molecular and cellular processes, from the recognition of specific *Lactobacillus* strains to IgA production in the intestine, have not been fully resolved.

In this study, we aimed to obtain cellular and molecular insights into the immunobiological activity of b240. Our results suggest that orally administered b240 was taken up by the subepithelial dome (SED) region of PPs and induced b240-Toll-like receptor (TLR) 2 axis-mediated the production of the proinflammatory cytokine IL-6 from PP DCs to activate B cells to produce a large amount of IgA.

## Materials and Methods

### Mice

BALB/c male mice were purchased from CLEA Japan, Inc. (Tokyo, Japan) or Japan SLC, Inc. (Hamamatsu, Japan) and were fed sterile food and water *ad libitum*. TLR2^−/−^, TLR4^−/−^, and TLR9^−/−^ male mice were originally established by Professor Shizuo Akira (Osaka University, Japan) [17]–[19] and genetically backcrossed 6 times for TLR2^−/−^ and TLR4^−/−^ mice and 8 times for TLR9^−/−^ mice with BALB/c mice at the experimental animal facility in our institute. All animals were used at 8–12 weeks of age for experiments. In the experiments using these gene knockout mice, we used the same mouse strain as used for the backcross. All animals were maintained in the experimental animal facility at the University of Tokyo, and the experiments were approved by the Animal Care and Use Committee of the University of Tokyo and conducted in accordance with their guidelines.

### Preparation of heat-killed b240

*Lactobacillus pentosus* strain b240 (ONRIC b0240; b240) was grown in a commercially available plant-based medium. The cultured b240 was washed twice with sterile saline to remove any metabolic substances, suspended in deionized water, and then autoclaved at 121°C for 15 min. The heat-killed bacterial suspension was lyophilized and stored at 4°C until use. Bacterial counts were determined using a flow cytometer as previously described [16]. One milligram of the lyophilized material contained 1.3×10^9^ counts of b240.

### Preparation of fluorescein isothiocyanate (FITC)-labeled b240

Heat-killed b240 was suspended at a concentration of 5 mg/ml in 50 mM carbonate buffer (pH 9.6; Sigma, St. Louis, MO, USA), reacted with FITC (Sigma) at 37°C for 60 min, washed twice with sterile Dulbecco's phosphate-buffered saline (D-PBS)(-) (Nacalai Tesque, Kyoto, Japan), and finally suspended in autoclaved water.

### Preparation of bacterial components

Bacterial cell components were prepared from heat-killed b240. Intact cell walls (ICW) of b240 were prepared according to the method described by Shida *et al*. [20] with minor modifications. Briefly, heat-killed b240 was suspended in a 0.3% solution of sodium dodecyl sulfate (Wako, Osaka, Japan) and boiled for 15 min. After centrifugation, the precipitate was washed with a 2∶1 mixture of methanol (Wako) and distilled water (Otsuka, Tokyo, Japan), methanol, and acetone (Wako). The b240 was treated with actinase E (Kaken, Tokyo, Japan) and delipidated with methanol, and a 1∶1 mixture of methanol and chloroform (Wako). The delipidated material was treated with DNase I (Sigma) and RNase A (Sigma), followed by treatment with actinase E. The insoluble material was washed with distilled water (Otsuka), lyophilized, and then used as ICW. To remove sugar from the ICW, the ICW was suspended in 2% potassium hydroxide (Wako) and boiled for 1 h. Potassium hydroxide-treated ICW was washed with distilled water and lyophilized, and then used as KOH-treated ICW. Neutral sugars in ICW and KOH-treated ICW were determined using the phenol-sulfuric acid method. Approximately 98% of the neutral sugars in the ICW were removed by KOH-treatment.

### Treatment regimes

To examine the distribution of orally administered b240 in the small intestine, mice were provided with autoclaved drinking water alone or supplemented with FITC-labeled b240 (1 mg/ml) *ad libitum* for 3 days, followed by histological analysis.

To examine the localization of b240 in PP, the ligated intestinal loop assay was conducted. Briefly, mice were anesthetized with isoflurane (Wako) and kept warm with a heat lamp during the assay. One hundred microliters of FITC-labeled heat-killed b240 in sterile D-PBS(-) (1 mg/ml) was injected into the ligated intestinal loop. After incubation for 3 h, the mice were killed and PPs were excised from the intestine, followed by histological analysis.

### Histological analysis

Histological analysis was performed as previously described [21] with minor modifications. Briefly, PPs were fixed in 4% paraformaldehyde (Nacalai Tesque), and treated with a sucrose gradient (10–20%). The tissue was embedded in Tissue-Tek optimal cutting temperature compound (Sakura Finetek, Torrance, CA, USA) and sliced into 7-μm-thick sections. The sections were stained with 4,6-diamidino-2-phenylindole (Wako) and/or biotinylated anti-CD11c antibody (HL3; BD Biosciences, Franklin Lakes, NJ, USA) using the TSA Plus Cyanine 3 System (PerkinElmer, Waltham, MA, USA) according to the manufacturer's instructions, and then analyzed using a fluorescence microscope (BZ-9000; Keyence, Osaka, Japan).

### Cell isolation

Cells were isolated from the PPs as previously described [21]. Briefly, PPs were removed and treated with collagenase (Wako) in RPMI-1640 (Nacalai Tesque) supplemented with 2% heat-inactivated newborn calf serum (Equitech-Bio, Kerrville, TX, USA) and 50 U/ml penicillin + 50 μg/ml streptomycin (Gibco, Carlsbad, CA, USA).

### Flow cytometry and cell sorting

Standard protocols were used for flow cytometric analysis and cell sorting as previously described [21]. Briefly, cells were first incubated with anti-CD16/32 antibody and then stained with fluorescent antibodies specific for B220 (RA3-6B2), CD4 (RM4-5), CD11c (HL3), CD19 (1D3), or IgD (11-26c.2a) (BD Biosciences). A Via-Probe solution (BD Biosciences) was used to distinguish between dead and live cells. Flow cytometric analysis and cell sorting were performed using the FACSCanto II and FACSAria systems (BD Biosciences), respectively. CD4^+^ cells as CD4^+^ T cells (approximately 95% purity), CD19^+^ cells as B cells (approximately 95% purity), and IgD^+^ cells as B cells (approximately 95% purity) were sorted from PP cells. For purification of DCs from the PP cells, B cells were depleted prior to staining using a magnetic-activated cell sorter (MACS) bead-conjugated antibody specific for B220 (Miltenyi Biotec, Bergisch Gladbach, Germany) using autoMACS (Miltenyi Biotec), and CD11c^+^B220^−^ cells were sorted as DCs (approximately 80% purity).

### Measurement of cytokines and IgA

The cytokine level in the culture supernatant was determined by the cytometric bead array (CBA) or enzyme-linked immunosorbent assay (ELISA). IL-4, IL-5, IL-6, IL-10, interferon (IFN)-γ, and tumor necrosis factor (TNF) were measured using the mouse Th1/Th2 cytokine or inflammation CBA kit (BD Biosciences). The detection limit for these cytokines was 20 pg/ml. BAFF and TGF-β1 levels were measured using Quantikine (R&D Systems, Minneapolis, MN, USA). IFN-α and IFN-β were measured using Mouse Interferon Alpha and Mouse Interferon Beta ELISA kit (PBL Biomedical Laboratories, Piscataway, NJ, USA), respectively. The detection limits for BAFF, TGF-β1, IFN-α, and IFN-β were 46.9, 31.2, 12.5, and 15.6 pg/ml, respectively.

Total IgA level in the culture supernatants was measured by ELISA as previously described [22]. The detection limit for IgA was 1.96 ng/ml.

### Culture medium

RPMI-1640 supplemented with 10% heat-inactivated fetal bovine serum (Gibco), 50 U/ml penicillin + 50 μg/ml streptomycin, 55 μM 2-mercaptoethanol (Gibco), and 1 mM sodium pyruvate (Gibco) were used in cell cultures as complete medium.

### PP cell culture

PP cells were cultured with or without heat-killed b240 in 1650 μl complete medium in a 24-well culture plate (Nunc, Penfield, NY, USA) (PP cells: 1.5 × 10^6^ cells/well, b240: 1.2×10^6^ or 1.2×10^7^ counts/well). The culture supernatants were collected on days 1, 3, 5, and 7 for the determination of cytokines and IgA.

PP cells were cultured with or without heat-killed b240 in 630 μl complete medium in a 48-well culture plate (PP cells: 5.8×10^5^ cells/well; b240: 4.7×10^6^ counts/well) in the presence or absence of 1 μM LE540 (Wako), which is an inhibitor of RA receptors, B cell maturation (BCMA)-Ig and transmembrane activator + CAML-interactor (TACI)-Ig (5 μg/ml each; these are Fc chimeras of the receptors of APRIL and BAFF; R&D Systems), neutralizing antibodies specific for IL-6 (MP5-20F3; anti-IL-6 mAb, 10 μg/ml; BD Biosciences), 10 μg/ml anti-IFN-γ mAb (R4-6A2; BD Biosciences), or 10 μg/ml anti-TNF mAb (MP6-XT3; BD Biosciences). Dimethyl sulfoxide (Wako) was used as the control for LE540, 10 μg/ml human IgG1 Fc antibody (R&D Systems) was used as the control for BCMA-Ig and TACI-Ig, and 10 μg/ml rat IgG1 k isotype control (R3-34; BD Biosciences) was used as the control for anti-IL-6 mAb, anti-IFN-γ mAb, and anti-TNF mAb. The culture supernatants were collected on day 4 for the determination of IgA.

PP cells were cultured with or without heat-killed b240 in 630 μl complete medium in a 48-well culture plate (BD Biosciences) (PP cells: 5.8×10^5^ cells/well, b240: 4.7×10^6^ counts/well) in the presence or absence of 0.4, 2, or 10 ng/ml recombinant IL-6 (rIL-6; BD Biosciences); 0.6, 3, or 15 ng/ml rIFN-γ (BD Biosciences); 0.08, 0.4, or 2 ng/ml rTNF (BD Biosciences). The culture supernatants were collected on day 4 for the determination of IgA.

### Sorted cell culture

CD11c^+^B220^−^ DCs, CD4^+^ T cells, and CD19^+^ B cells were sorted from the PPs, then each cell type or PP cells (1×10^5^ cells/well) were cultured with or without heat-killed b240 (1.6×10^6^ counts/well) in 210 μl complete medium in a 96-well culture plate (Nunc) for 3 days to determine the IL-6 concentration in the supernatants.

CD11c^+^B220^−^ DCs (1×10^5^ cells/well) and/or IgD^+^ B cells (2×10^5^ cells/well) sorted from the PPs of wild type (WT) or TLR2^−/−^ mice were cultured with or without heat-killed b240 (1.6×10^6^ counts/well) in 250 μl complete medium in a 96-well culture plate in the presence or absence of 10 μg/ml anti-IL-6 mAb for 7 days to determine the concentration of IgA and IL-6 in the supernatants.

### Microscopic analysis of the interaction between PP DCs and b240

CD11c^+^B220^−^ DCs (1×10^5^ cells/well) were sorted from the PPs and cultured with FITC-labeled b240 (1.6×10^6^ counts/well) on a Cell Desk LF (Sumitomo Bakelite, Tokyo, Japan) inserted into a 24-well culture plate for overnight. The desk was washed twice with D-PBS(-), fixed in 4% paraformaldehyde, washed twice with D-PBS(-), and then analyzed using a fluorescence microscope.

### Statistics

All results were expressed as mean ± SEM. Unpaired *t*-test, one-way analysis of variance (ANOVA) (Dunnett), or two-way ANOVA (Dunnett) were used to compare the differences between groups. A two-tailed *P*-value<0.05 was accepted as significant for all tests. Data were analyzed using SAS software R9.1 (SAS Institute, Cary, NC, USA).

## Results

### Orally administered b240 interacts with PP cells

**Figure 1:**
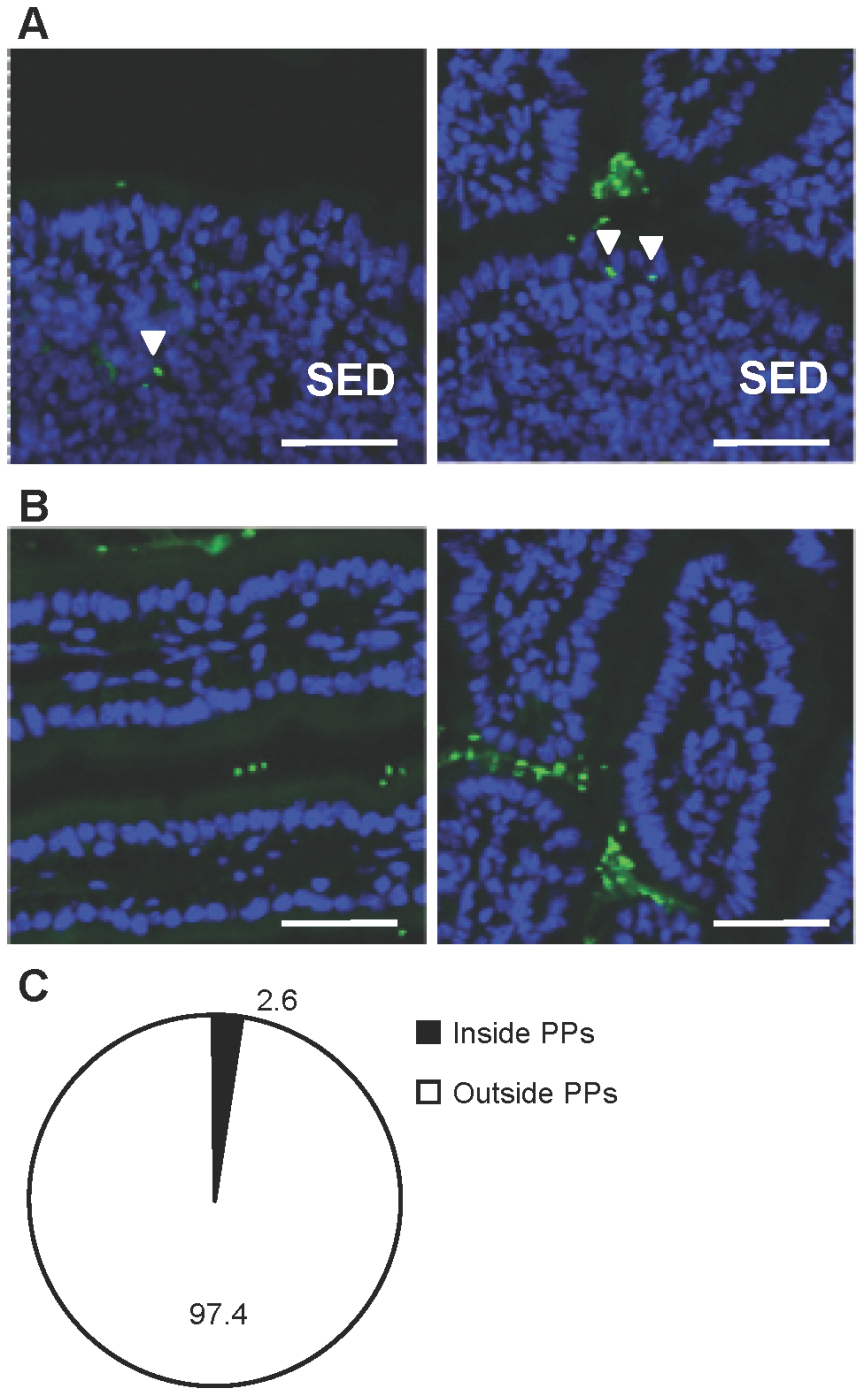
Localization of orally administered b240 in the small intestine. Histological analysis was performed to examine the localization of b240 in (A) the PPs and (B) the small intestine after mice were provided with autoclaved drinking water supplemented with 1 mg/ml of FITC-labeled b240 *ad libitum* for 3 days. Tissue sections were prepared and then stained with 4,6-diamidino-2-phenylindole (blue). b240 (green) internalized in PP are indicated by arrowheads. The bar indicates 50 μm. (C) The number of b240 inside PPs and in the luminal areas of the tissue sections was counted. Then, the proportion of b240 inside the PPs was calculated. Data are representative of 2 independent experiments producing similar results.

In our previous study, orally administered heat-killed b240 was confirmed to promote IgA production *in vitro* using PP cells [14]. We thus first tested whether heat-killed b240 is transported into PPs *in vivo*. Histological analysis of the small intestine after oral administration of FITC-labeled b240 in mice for 3 days revealed that b240 or a component of b240 was taken up into the SED region of the PPs (Figure 1A), while relatively little b240 or a component of b240 was found inside the small intestinal lamina propria (Figure 1B). Furthermore, we measured the proportion of b240 or a component of b240 inside PPs and in the lumen, showing that 2.6% of the b240 or a component of b240 was inside PPs (Figure 1C). Our previous and current findings collectively suggest that b240 or a component of b240 is taken directly up by the PPs and stimulates PP immunocompetent cells to initiate IgA production.

### b240 promotes IgA production from PP cells by enhancing IL-6 production

**Figure 2:**
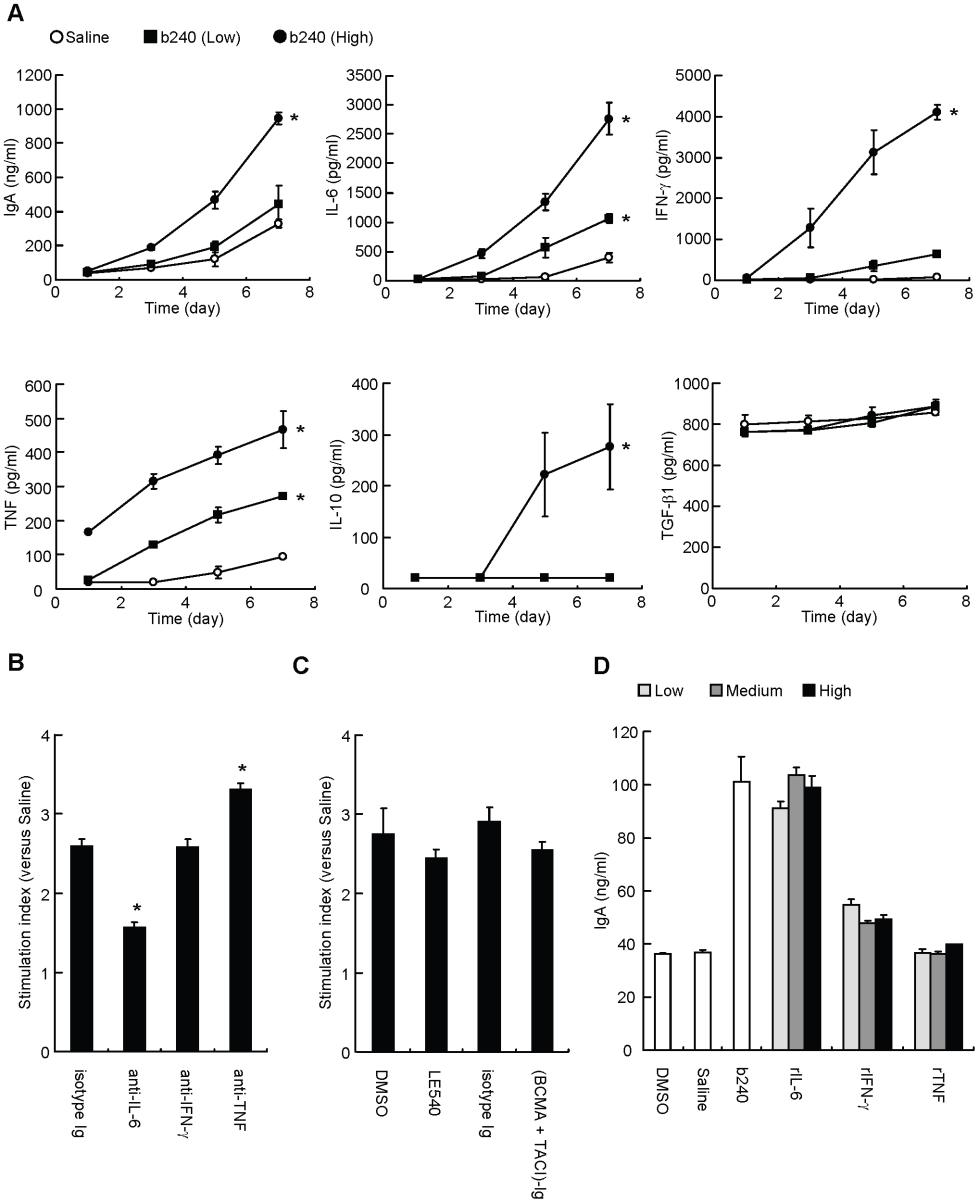
Important factor for the enhancement IgA production from PP cells by b240. (A) PP cells (1.5×10^6^ cells) were cultured with saline (open circles), 1.2×10^6^ counts of heat-killed b240 (closed squares), or 1.2 × 10^7^ counts of heat-killed b240 (closed circles) for 1, 3, 5, and 7 days. (B, C) In the presence or absence of heat-killed b240 (4.7×10^6^ counts), PP cells (5.8×10^5^ cells) were cultured with (B) anti-IL-6 mAb (10 μg/ml), anti-IFN-γ mAb (10 μg/ml), anti-TNF mAb (10 μg/ml), rat IgG1 k isotype control (10 μg/ml), (C) LE540 (1 μM), BCMA-Ig+ TACI-Ig (5 μg/ml each), dimethyl sulfoxide, or human IgG1 Fc antibody (10 μg/ml) for 4 days. The stimulation index of each sample was calculated (for example, (b240-treatment and anti-IL-6 Ab treatment)/(saline-treatment and anti-IL-6 Ab treatment) is the stimulation index for anti-IL-6 Ab treatment). (D) PP cells (5.8×10^5^ cells) were cultured with a low dose (light gray), medium dose (dark gray), and high dose (black) of rIL-6 (0.4, 2, or 10 ng/ml), rIFN-γ (0.6, 3, or 15 ng/ml), rTNF (0.08, 0.4, or 2 ng/ml), or heat-killed b240 (4.7×10^6^ counts) for 4 days. IgA or cytokine in the culture supernatants was determined by ELISA or CBA. Data are expressed as mean ± SEM (n = 3). (A, B) ^*^ *P*<0.05 versus control group by Dunnett's test. (C) Student's *t*-test was conducted. (D) Statistical analysis was not conducted. Data are representative of 2 independent experiments producing similar results.

The direct evidence of b240 uptake in PP tissue prompted us to examine the molecular network underlying b240-mediated and b240-enhanced IgA production by PP cells using an *in vitro* system. When PP cells were cultured with saline or heat-killed b240 for 1, 3, 5, and 7 days, b240 was found to promote the production of IgA, IL-6, IL-10, IFN-γ, and TNF, but not TGF-β1 (Figure 2A). In contrast, IL-4, IL-5, soluble BAFF, IFN-α, and IFN-β were not detected. IL-6 and IFN-γ production promoted by b240 were kinetically synchronous with IgA production. In contrast, TNF was produced prior to the production of IL-6 and IFN-γ, and IL-10 was produced subsequent to the production of these 2 cytokines (Figure 2A). To examine whether these cytokines are involved in enhanced IgA production, PP cells were cultured with b240 in the presence of blocking antibodies specific for IL-6, IFN-γ, or TNF. Enhanced IgA production by b240 was attenuated by the neutralization of IL-6, but not IFN-γ and TNF (Figure 2B and Table S1A). Reciprocally, exogenous rIL-6, but not rIFN-γ and rTNF, promoted IgA production by PP cells (Figure 2D). In addition, we confirmed that inhibition of soluble and membrane-bound APRIL, membrane-bound BAFF, and RA, all of which are factors closely associated with IgA production, did not affect the b240-mediated production of IgA (Figure 2C and Table S1B). These results suggest that IL-6 induced by b240 is mainly involved in enhancing IgA production from PP cells.

### b240 promotes IL-6 production from PP cells through TLR2

**Figure 3:**
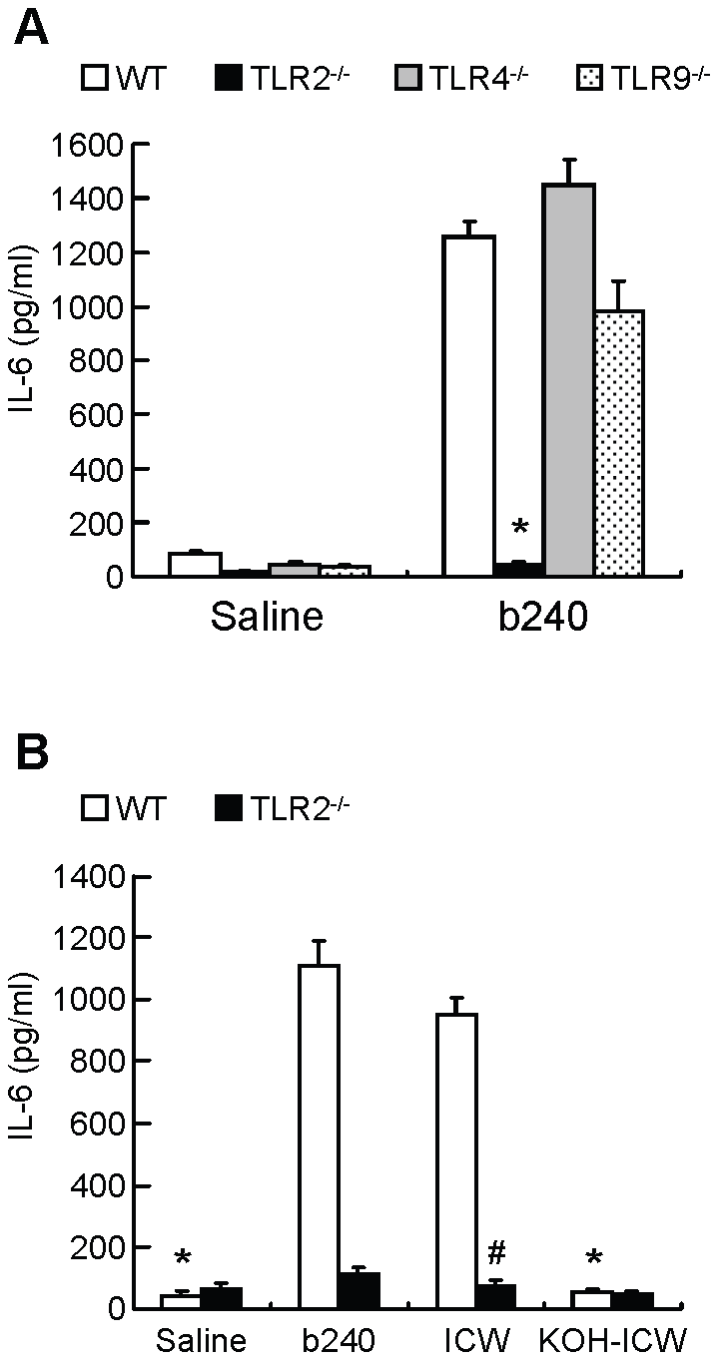
Involvement of TLR signals in the IL-6-inducing ability of b240. (A) PP cells (5.8×10^5^ cells) from WT (white), TLR2^−/−^ (black), TLR4^−/−^ (gray), or TLR9^−/−^ (dotted) mice were cultured with or without heat-killed b240 (4.7×10^6^ counts) for 4 days. IL-6 in the culture supernatants was determined by CBA. ^*^ *P*<0.05 versus WT in b240 group by Dunnett's test. The saline group was not tested. (B) PP cells (5.8×10^5^ cells) from WT (white) or TLR2^−/−^ (black) mice were cultured with or without heat-killed b240 (4.7×10^6^ counts), the intact cell wall (ICW), or the KOH-treated ICW for 4 days. The ICW and the KOH-treated ICW were applied in the same amount as heat-killed b240. IL-6 in the culture supernatants was determined by CBA. ^*^ *P*<0.05 versus b240 in the WT group by Dunnett's test, and the TLR2^−/−^ group was not tested. ^#^ *P*<0.05 versus WT in the ICW group by Student's *t*-test. Data are expressed as mean ± SEM (n = 3–6). Data are (A) combined or (B) representative of 2 independent experiments.

We next tried to identify the receptors on PP cells engaged in IL-6 induction by b240. TLRs are critical in triggering innate immune responses such as proinflammatory cytokine (e.g., TNF and IL-6) production for the initiation of the immune response [37]. TLR2, 4, and 5 recognize the conserved structures of bacterial membrane components (e.g., lipids and lipoproteins) and TLR9 recognizes that of DNA [37]. In this study, we examined whether TLR2, 4, or 9 are involved in enhanced IL-6 production by b240. To address this issue, PP cells from WT, TLR2^−/−^, TLR4^−/−^, or TLR9^−/−^ mice were cultured with or without b240. Even in the presence of b240, IL-6 production from TLR2^−/−^, but not TLR4^−/−^ and TLR9^−/−^, PP cells was substantially decreased (Figure 3A). We also examined whether TLR2, 4, and 9 are functional in PP cells from WT mice using reverse transcription polymerase chain reaction (RT-PCR) and a PP cell culture system. WT PP cells expressed TLR2, 4, and 9 specific mRNA (Figure S2A and Supporting information S1) and produced IL-6 in response to TLR2, 4, and 9 ligands (Figure S1 and Supporting information S1). Furthermore, to examine the effect of b240 on PP cell viability, WT PP cells were cultured with or without b240. The cell viability of b240-stimulated PP cells was about 1.5-fold higher than that of control (Figure S3). These results suggest that these TLRs work properly in the PP cells and that TLR2 recognition of b240 contributes to enhancing IL-6 production in PP cells mainly by enhancing the immune response, but not by maintaining cell viability.

To examine the component responsible for IL-6 induction via TLR2, ICW of b240 and sugar-removed ICW (KOH-treated ICW) were prepared and examined using the PP cell culture system. ICW, but not sugar-removed ICW, promoted IL-6 production comparable to whole b240. As expected, ICW failed to promote IL-6 production from TLR2^−/−^ PP cells (Figure 3B). These results suggest that the polysaccharide-peptidoglycan portion of b240 promotes IL-6 production from PP cells through TLR2.

### Co-localization of mucosally administered b240 with PP DCs is associated with elevated IL-6 production

**Figure 4:**
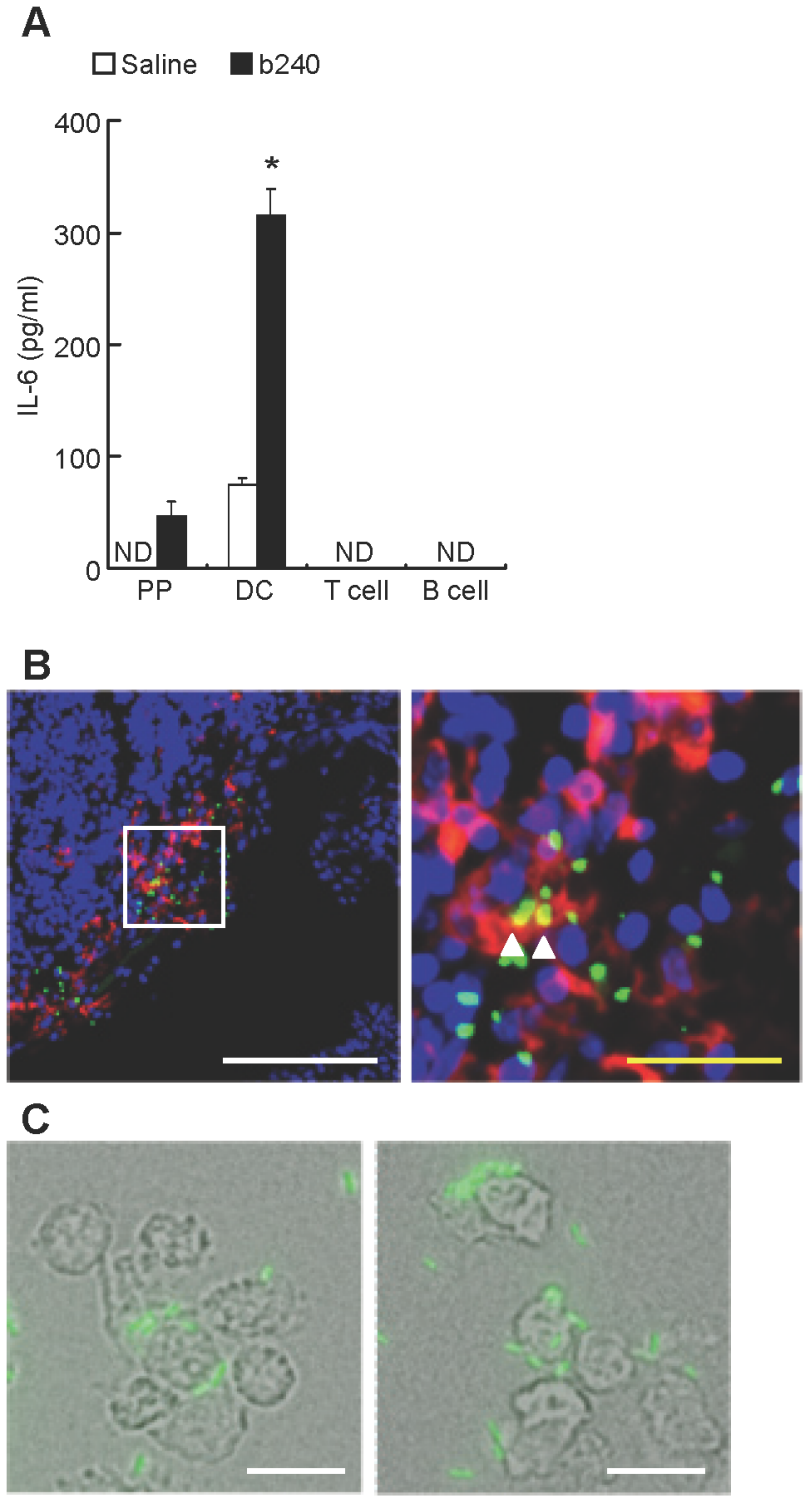
IL-6 producing cells in the PPs in response to b240. (A) Purified CD11c^+^B220^−^ cells as DCs, CD4^+^ cells as T cells, CD19^+^ cells as B cells from the PPs, or PP cells (1×10^5^ cells) were cultured with (black) or without (white) heat-killed b240 (1.6×10^6^ counts) for 3 days. IL-6 in the culture supernatants was determined by CBA. Data are expressed as mean ± SEM (n = 3). ND, not detected. The detection limit was 20 pg/ml. ^*^ *P*<0.05 by Student's *t*-test. Data are representative of 2 independent experiments producing similar results. (B) After the ligated intestinal loop assay with 1 mg/ml of FITC-labeled b240, histological analysis was performed to examine the co-localization of b240 (green) with DCs in PP. Tissue sections were prepared and stained with anti-CD11c antibody (red) and 4,6-diamidino-2-phenylindole (blue). Magnification of the area in the first image (white square) shows the contact between b240 and CD11c^+^ cells (arrowhead). White and yellow bars indicate 100 and 25 μm, respectively. (C) After PP CD11c^+^B220^−^ DCs were cultured with FITC-labeled b240 on a Cell Desk, the interaction between PP CD11c^+^B220^−^ DCs and b240 was analyzed using a fluorescence microscope. The bar indicates 10 μm.

To examine which cells in PPs are responsible for TLR2-mediated IL-6 production in response to b240, CD11c^+^B220^−^ DCs, CD4^+^ T cells, or CD19^+^ B cells were purified from PPs and then cultured with b240. Among them, only PP DCs were observed to secrete a large amount of IL-6 in response to b240, whereas neither B nor T cells produced IL-6 in response to b240 (Figure 4A). We also examined whether TLR2, 4, and 9 are expressed and functional in each purified cell type. To address this issue, the expression levels of TLR2, 4, and 9 mRNA in each purified cell type were examined by RT-PCR and each purified cell type was cultured with TLR2 ligand. CD11c^+^B220^−^ DCs, CD4^+^ T cells, and CD19^+^ B cells expressed TLR2, 4, and 9 mRNA (Figure S2A and Supporting information S1): however; CD11c^+^B220^−^ DCs and CD19^+^ B cells, but not CD4^+^ T cells, produced IL-6 in response to TLR2 ligand (Figure S2B and Supporting information S1). In this issue, we confirmed that isolated CD4^+^ T cells were alive and functional because CD4^+^ T cells produced IL-2 upon stimulation by anti-CD3 and anti-CD28 antibodies (Figure S2C and Supporting information S1). These results suggest that purified cells from PPs are viable and CD11c^+^B220^−^ DCs and CD19^+^ B cells expressed functional TLR2, and that CD11c^+^B220^−^ DCs are responsible for IL-6 production in response to b240 in PPs.

To examine whether b240 comes in contact with DCs in PPs, we conducted a ligated intestinal loop assay with FITC-labeled b240. Histological analysis revealed that b240 co-localized with CD11c^+^ cells in the SED region of the PPs (Figure 4B). Furthermore, microscopic analysis was performed to evaluate the interaction between PP DCs and b240. We found that FITC-labeled b240 mainly contacted the cell surface of PP DCs (Figure 4C). Thus, it is plausible that DCs recognize b240 in PPs and contribute to b240-promoted IgA production by promoting IL-6 production.

### TLR2-mediated IL-6 production by PP DCs is required for enhanced IgA production

**Figure 5:**
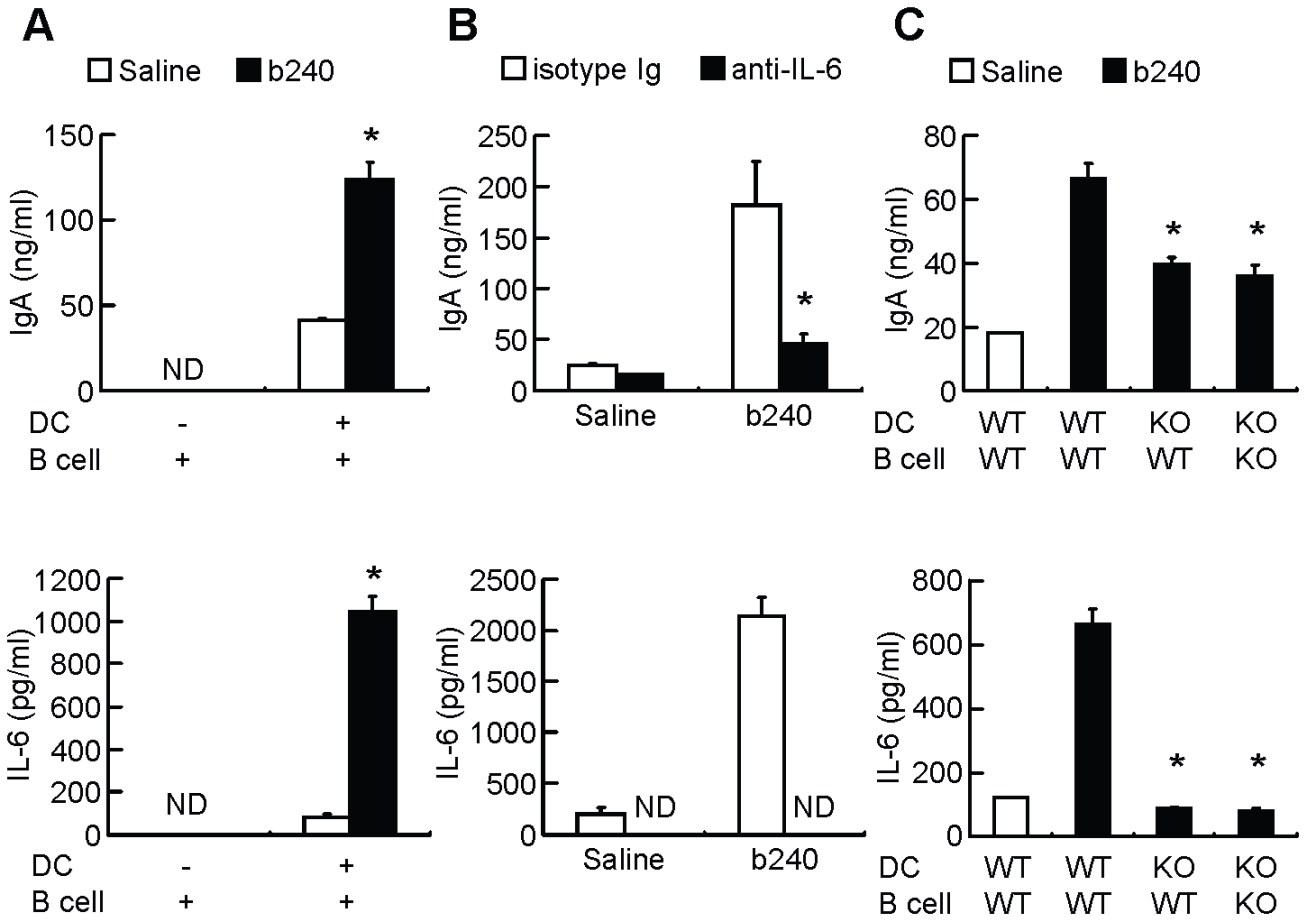
Role of PP DCs in the IgA-enhancing ability of b240. (A, C) In the presence (black) or absence (white) of heat-killed b240 (1.6×10^6^ counts), (A) purified WT PP IgD^+^ cells (2×10^5^ cells/well) were cultured with or without purified WT PP CD11c^+^B220^−^ DCs (1×10^5^ cells/well), (B) purified WT PP CD11c^+^B220^−^ DCs and purified WT PP IgD^+^ B cells were co-cultured with (black) or without (white) anti-IL-6 mAb (10 μg/ml) in the presence or absence of heat-killed b240 (1.6×10^6^ counts), and (C) purified WT or TLR2^−/−^ PP CD11c^+^B220^−^ DCs and purified WT or TLR2^−/−^ PP IgD^+^ B cells were co-cultured for 7 days. IgA and IL-6 in the culture supernatants were determined by ELISA and CBA, respectively. Data are expressed as mean ± SEM (n = 1–3). ND, not detected. ^*^ *P*<0.05 versus (A) saline group by Welch's *t*-test, (B) isotype Ig group by Student's t-*t*est, (C) WT DCs + WT B cells + b240 co-culture by Dunnett's test. Data are representative of 2 independent experiments producing similar results.

To confirm whether IL-6 production by b240-stimulated PP CD11c^+^B220^−^ DCs is responsible for enhanced IgA production, PP CD11c^+^B220^−^ DCs and/or PP IgD^+^ B cells were cultured with or without b240. In the presence of PP CD11c^+^B220^−^ DCs and PP IgD^+^ B cells, b240 induced high amounts of IgA and IL-6 in the culture supernatants, whereas in the absence of PP CD11c^+^B220^−^ DCs and the presence of PP IgD^+^ B cells alone, b240 failed to induce IgA (Figure 5A). As observed in the PP cell culture system, enhanced IgA production due to IL-6 production from b240-stimulated PP CD11c^+^B220^−^ DCs was decreased by anti-IL-6 mAb treatment (Figure 5B). However, when PP IgD^+^ B cells were cultured with b240 and IL-6, IgA production was hardly enhanced (Figure S4 and Supporting information S1). Finally, we examined whether TLR2-mediated IL-6 production from PP CD11c^+^B220^−^ DCs in response to b240 contributes to enhancing IgA production from PP IgD^+^ B cells using a co-culture system with WT or TLR2^−/−^ PP CD11c^+^B220^−^ DCs and PP IgD^+^ B cells. We found that TLR2^−/−^ PP CD11c^+^B220^−^ DCs failed to produce IL-6, and that the subsequent IgA production from either WT or TLR2^−/−^ PP IgD^+^ B cells upon b240 stimulation was partially reduced (Figure 5C). These results suggest that TLR2-mediated recognition of b240 by PP CD11c^+^B220^−^ DCs, but not by PP IgD^+^ B cells, is critical for the IL-6-enhancing ability of b240 and plays a relevant, albeit partial, role in the IgA-enhancing ability of b240. There might be additional factors produced by PP DCs that assist in PP B cell IgA production.

## Discussion

In the present study, we continuously used heat-killed b240 for molecular and cellular understanding of “immunobiotics” activity based on our previous results that the IgA-enhancing ability of heat-killed b240 is comparable to that of live b240 *ex vivo* [14]. Our present study showed that b240 co-localized with DCs in the SED region of the PPs after administration of b240 into an intestinal ligated loop, and TLR2-mediated IL-6 production from PP DCs led to the activation of B cells to produce a large amount of IgA in an *in vitro* culture system.

It has been suggested that specific *Lactobacillus* spp. induce immune responses via the following 3 pathways [30]. First, they translocate into PPs through M cells and affect the resident antigen-presenting cells in the PPs, including DCs and macrophages in the SED region. Second, they stimulate epithelial cells to produce humoral factors such as thymic stromal lymphopoietin and APRIL. Third, DCs in the lamina propria extend their dendrites into the gut lumen to sample the bacteria. It was shown that a specific *Lactobacillus* strain was found only in the PPs, whereas some *Lactobacillus* strains were observed in both PPs and the small intestinal villi when they were orally administered to mice [31]. These observations suggest that there is an intestinal site specificity of strains in the internalization of orally administered *Lactobacilli* in the intestine. In the present study, we detected that orally administered b240 was localized preferentially in the PPs. Thus, although we cannot exclude the possibility that b240 affects intestinal epithelial cells or DCs in the small intestinal lamina propria, our current findings indicate that it is plausible that b240 is primarily taken up by and present in the PPs, where it stimulates DCs to enhance IL-6 production and the subsequent IgA production. Additionally, the green observed in intestinal tissue sections is believed to be whole b240, but the possibility of interaction with a degraded component of b240 cannot be excluded.

The relevant role of IL-6 in b240-mediated IgA enhancement is further confirmed in the DC-B cell *in vitro* co-culture system (Figure 5B). It was reported that IL-6 markedly promotes IgA production from IgA-committed B cells [35] and that disruption of the gene encoding IL-6 leads to poor IgA responses in the intestine and lungs [36]. These lines of evidence rationalize the hypothesis that b240 promotes the differentiation of IgA^+^ B cells into IgA-producing cells by enhancing IL-6 production from PP DCs. We also suggest that APRIL, BAFF, and RA are not involved in enhancing IgA production by b240 from PP cells, since the simulation of PP DCs with b240 did not induce APRIL, BAFF, and RA production (Figure 2C). Although TLR2^−/−^ PP DCs failed to produce IL-6 in response to b240, b240 partially induces IgA production in TLR2^−/−^ PP DC-TLR2^−/−^/WT PP B cell co-culture (Figure 5C). In addition, IgA production was hardly enhanced when PP B cells were cultured with b240 and IL-6. These findings suggest that an unidentified additional receptor other than TLR2 on PP DCs may be involved in the IgA-enhancing ability of b240 through certain factors other than APRIL, BAFF, and RA. Further experiments will be needed to identify other factors participating in B cell IgA production.

There are few reports investigating immune responses to a particular component of specific *Lactobacillus* strains through the TLRs of host cells. TLRs are pattern-recognition receptors that recognize molecular structures broadly shared by pathogens [37]. TLR2 recognizes bacterial-specific cell wall components such as peptidoglycan and lipoteichoic acid [37]. In the present study, we showed that the polysaccharide-peptidoglycan portion of b240 was critical in inducing IL-6 production from PP cells through TLR2. It was reported that the ICW of *Lactobacillus casei* strain Shirota was capable of inducing IL-12 production from peritoneal macrophages, but that polysaccharide-removed ICW was not [33]. These observations indicate that the polysaccharide-peptidoglycan portion of specific *Lactobacillus* strains is one of the indispensible players for their immunomodulatory effects.

Murine PP DCs express TLR1, 2, 4, 6, 7, 8, and 9 mRNA [38] and can be divided into 3 subsets: CD11b^+^CD8α^−^ DCs (CD11b^+^ DCs), CD11b^−^CD8α^+^ DCs, and CD11b^-^CD8α^−^ DCs [39]. Among them, CD11b^+^ DCs predominantly secrete IL-6 [40] and reside in the SED region of the PPs [39]. In the present study, we demonstrated that b240 co-localized with CD11c^+^ cells in the SED region of the PP by a ligated intestinal loop assay, indicating that CD11b^+^ DCs in the SED region of the PP produce IL-6 in response to b240 *in vivo*.

Here, we have confirmed that b240 co-localized with DCs in the SED region of PPs induces TLR2-mediated IL-6 production from PP DCs and results in the activation of B cells to produce a large amount of IgA. Thus, the well-characterized immunological nature of b240 will pave the way for the wide recognition of b240 as a useful prospective immunobiotic contributing to the promotion of health by enhancing IgA production in the intestine.

## Supporting Information

Supporting information S1 **Materials, Methods, and References.**
(DOC)

Table S1 **IgA production from non-stimulated PP cells.** (A, B) In the absence of heat-killed b240 (4.7×10^6^ counts), PP cells (5.8×10^5^ cells) were cultured with (A) anti-IL-6 mAb (10 μg/ml), anti-IFN-γ mAb (10 μg/ml), anti-TNF mAb (10 μg/ml), rat IgG1 k isotype control (10 μg/ml), (B) LE540 (1 μM), BCMA-Ig+ TACI-Ig (5 μg/ml each), dimethyl sulfoxide, or human IgG1 Fc antibody (10 μg/ml) for 4 days. IgA concentrations in the culture supernatants were determined by ELISA. Data are expressed as mean ± SEM (n = 3). Data are representative of 2 independent experiments producing similar results.
(TIF)

Figure S1 **IL-6 production from TLR2, 4, or 9-stimulated PP cells.** PP cells (5.8×10^5^ cells) were cultured with or without heat-killed b240 (4.7×10^6^ counts), Pam_3_CSK_4_ (1 μg/ml), LPS (1 μg/ml), or ODN 1826 (1 μg/ml) for 4 days. IL-6 concentrations in the culture supernatants were determined by cytometric bead array. Data are expressed as mean ± SEM (n = 3).
(TIF)

Figure S2 **TLR expression and function in CD11c^+^B220^−^ DCs, CD4^+^ T cells, and CD19^+^ B cells from PPs.** (A) Purified CD11c^+^B220^−^ DCs, CD4^+^ T cells, CD19^+^ B cells from PPs, and PP cells, were analyzed for gene expression levels of tlr2, 4, and 9. Expression was determined as fold induction compared with the β-actin housekeeping gene. Data are expressed as mean ± SD (n = 3). (B) Purified CD11c^+^B220^−^ DCs, CD4^+^ T cells, or CD19^+^ B cells (1×10^5^ cells) from the PPs were cultured with or without Pam_3_CSK_4_ (1 μg/ml) in a 96-well flat-bottomed plate for 3 days and then IL-6 concentrations in the culture supernatants were determined by cytometric bead array (CBA). (C) Purified CD4^+^ T cells (1×10^5^ cells) from the PPs were cultured with or without pre-coated anti-CD3 antibody and anti-CD28 antibody (1 μg/ml) in a 96-well round-bottomed plate for 3 days, and then IL-2 concentrations in the culture supernatants were determined by CBA. (B, C) Data are expressed as mean ± SEM (n = 3).
(TIF)

Figure S3 **The effect of b240 on the PP cell viability.** PP cells (5.8×10^5^ cells) were cultured with or without heat-killed b240 (4.7×10^6^ counts) for 4 days and then cell viability was evaluated by the Trypan blue dye exclusion test. Data are expressed as mean ± SEM (n = 5).
(TIF)

Figure S4 **IgA production from b240- and IL-6-treated PP B cells.** PP IgD^+^ B cells (2×10^5^ cells) were cultured with or without PP CD11c^+^B220^−^ DCs (5×10^4^ cells), heat-killed b240 (1.6×10^6^ counts), or rIL-6 (0.2, 1.0, 5.0 ng/ml) for 7 days, and then IgA concentrations in the supernatants were determined by ELISA. The x-axis indicates the detection limit for IgA.
(TIF)
