# Supplementary material for: Role of Lactobacillus pentosus Strain b240 and the Toll-Like Receptor 2 Axis in Peyer's Patch Dendritic Cell-Mediated Immunoglobulin A Enhancement
Source: PLoS One. 2014 Mar 14;9(3):e91857. doi: 10.1371/journal.pone.0091857 (PMC3954862; doi:10.1371/journal.pone.0091857)
Supplement: Supporting information S1 — Materials, Methods, and References. (DOC) [file pone.0091857.s001.doc]

**Supporting information S1. Materials, Methods, and References**

**Reagents**

Pam_3_CSK_4_ and ODN 1826 were purchased from InvivoGen (San Diego, CA, USA). Lipopolysaccharide (LPS) from *E. coli* 055:B5 was purchased from Sigma (St. Louis, MO, USA). Purified anti-mouse CD3ε antibody (clone 145-2C11) was purchased from BioLegend (San Diego, CA, USA). Purified anti-mouse CD28 antibody (clone 37.51) was purchased from BD Biosciences (Franklin Lakes, NJ, USA).

**PP cell culture**

PP cells were cultured with or without heat-killed b240 in 630 μl complete medium in a 48-well culture plate (BD Biosciences) (PP cells: 5.8 × 10^5^ cells/well, b240: 4.7 × 10^6^ counts/well) in the presence or absence of 1 μg/ml Pam_3_CSK_4_ (InvivoGen); 1 μg/ml LPS (Sigma); 1 μg/ml ODN 1826 (InvivoGen). The culture supernatants were collected on day 4 for determination of IL-6 levels.

**Sorted cell culture**

CD11c^+^B220^−^ DCs, CD4^+^ T cells, and CD19^+^ B cells were sorted from the PPs, then each cell type (1 × 10^5^ cells/well) was cultured with or without Pam_3_CSK_4_ (1 μg/ml) in 210 μl complete medium in a 96-well flat-bottomed culture plate (Nunc, Penfield, NY, USA) for 3 days to determine IL-6 concentrations in the supernatants.

A 96-well round-bottomed culture plate (Nunc) was coated with 2.5 μg/ml anti-CD3 antibody at 4°C overnight and the antibody solution was removed. Then, CD4^+^ T cells were cultured with 1 μg/ml anti-CD28 antibody to determine IL-2 levels.

IgD^+^ B cells and CD11c^+^B220^−^ DCs were sorted from the PPs and then IgD^+^ B cells (2 × 10^5^ cells) were cultured with or without CD11c^+^B220^−^ DCs (5 × 10^4^ cells), heat-killed b240 (1.6 × 10^6^ counts), or rIL-6 (0.2, 1.0, 5.0 ng/ml) for 7 days to determine IgA concentrations in the supernatants.

**Real-time quantitative reverse transcription polymerase chain reaction (RT-PCR)**

Total RNA was extracted from purified CD11c^+^B220^−^ DCs, CD4^+^ T cells, and CD19^+^ B cells from the PPs, and from PP cells, for RT-PCR as previously described [1] with minor modifications. Briefly, Sepasol (Nacalai Tesque) was used for RNA extraction. Complementary DNA (cDNA) transcripts were generated using the VILO cDNA synthesis kit (Invitrogen, Carlsbad, CA, USA). RT-PCR was performed using LightCycler 480 II (Roche Diagnostics GmbH, Mannheim, Germany). Primers for TLR2, 4, and 9 were used as reported previously [2]. β-actin primers were used for the control. The sequences of the primers used were as follows: tlr2, (sense) 5′-ggggcttcacttctctgctt-3′ and (anti-sense) 5′- agcatcctctgagatttgacg-3′; tlr4, (sense) 5′- ggactctgatcatggcactg-3′ and (anti-sense) 5′- ctgatccatgcattggtaggt -3′; tlr9, (sense) 5′- gaatcctccatctcccaacat-3′ and (anti-sense) 5′- ccagagtctcagccagcact-3′; β-actin, (sense) 5′- ctaaggccaaccgtgaaaag-3′, and (anti-sense) 5′- accagaggcatacagggaca-3′. Gene expression levels were calculated relative to β-actin.

**References**

1. Shikina T, Hiroi T, Iwatani K, Jang MH, Fukuyama S, et al. (2004) IgA class switch occurs in the organized nasopharynx- and gut-associated lymphoid tissue, but not in the diffuse lamina propria of airways and gut. J Immunol 172: 6259-6264.
2. Davies JM, MacSharry J, Shanahan F. (2010) Differential regulation of Toll-like receptor signaling in spleen and Peyer’s patch dendritic cells. Immunology 131: 438-448.
