# Supplementary figures and images for: Role of Lactobacillus pentosus Strain b240 and the Toll-Like Receptor 2 Axis in Peyer's Patch Dendritic Cell-Mediated Immunoglobulin A Enhancement
Source: PLoS One. 2014 Mar 14;9(3):e91857. doi: 10.1371/journal.pone.0091857 (PMC3954862; doi:10.1371/journal.pone.0091857)

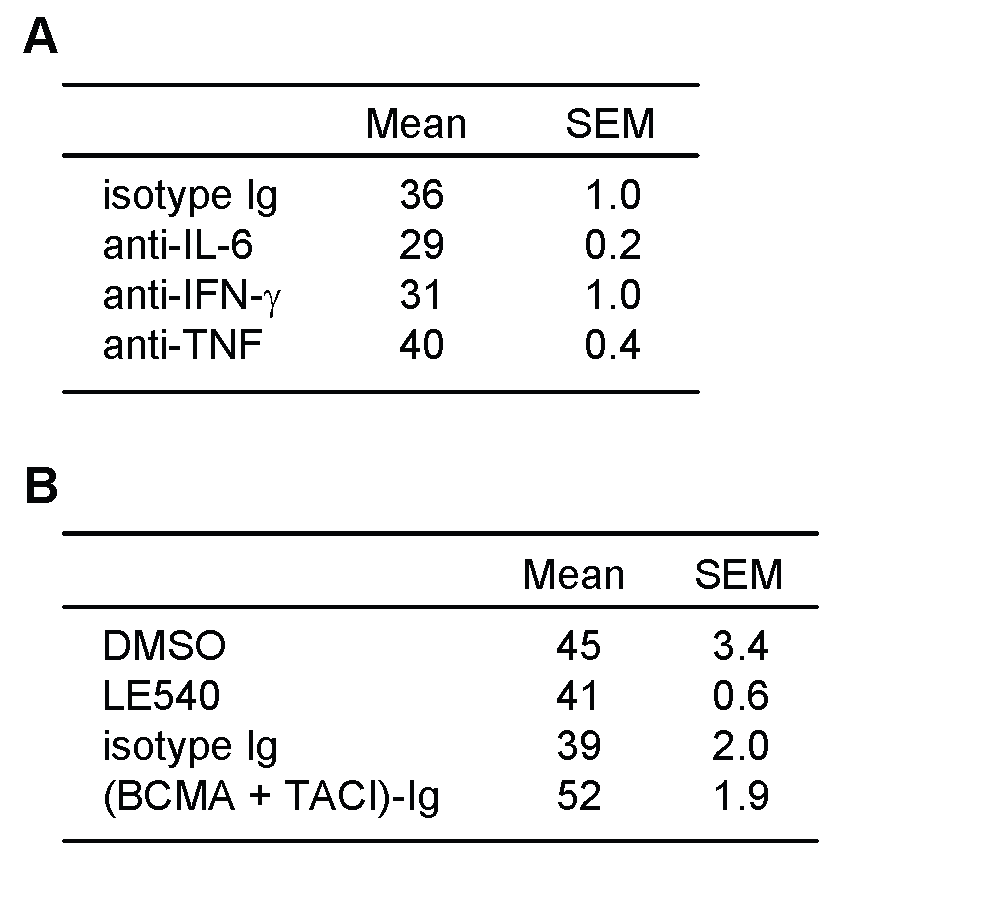

Supplement: Table S1 — IgA production from non-stimulated PP cells. (A, B) In the absence of heat-killed b240 (4.7×10^6^ counts), PP cells (5.8×10^5^ cells) were cultured with (A) anti-IL-6 mAb (10 μg/ml), anti-IFN-γ mAb (10 μg/ml), anti-TNF mAb (10 μg/ml), rat IgG1 k isotype control (10 μg/ml), (B) LE540 (1 μM), BCMA-Ig+ TACI-Ig (5 μg/ml each), dimethyl sulfoxide, or human IgG1 Fc antibody (10 μg/ml) for 4 days. IgA concentrations in the culture supernatants were determined by ELISA. Data are expressed as mean ± SEM (n = 3). Data are representative of 2 independent experiments producing similar results. (TIF) [file pone.0091857.s002.tif]

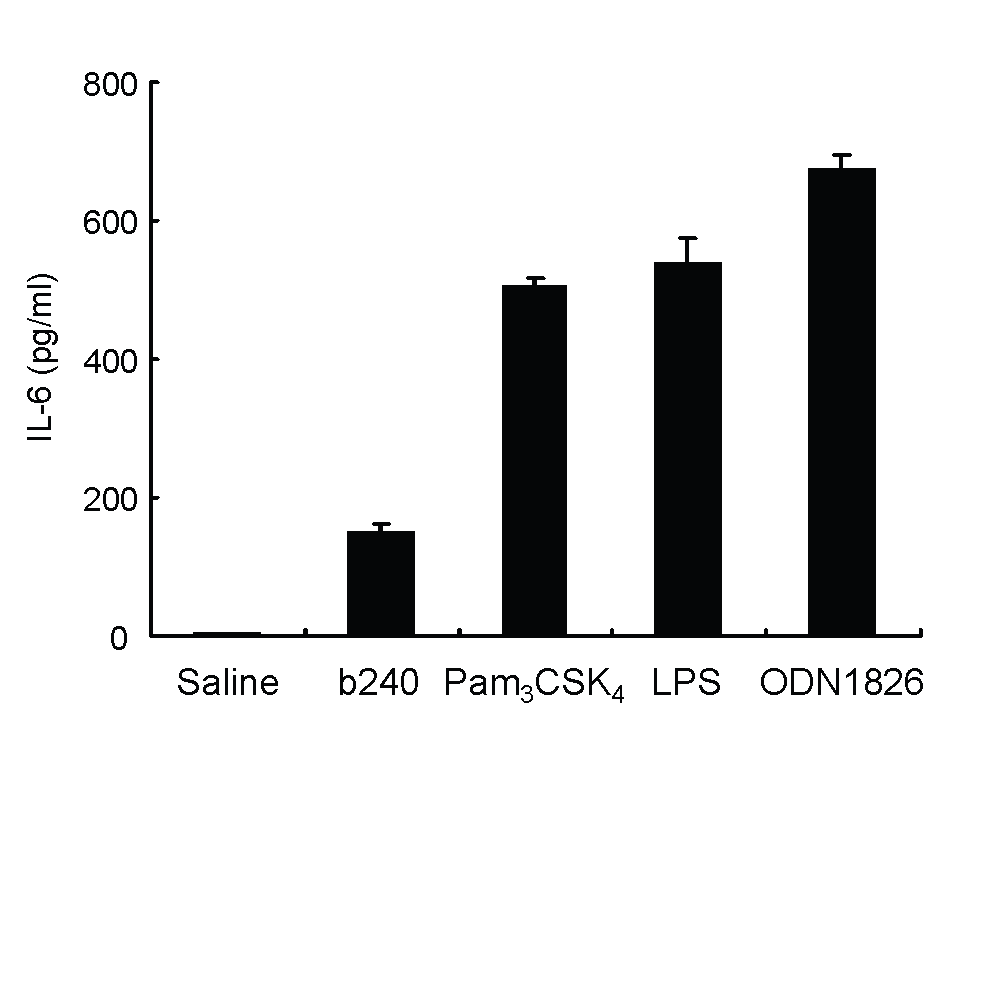

Supplement: Figure S1 — IL-6 production from TLR2, 4, or 9-stimulated PP cells. PP cells (5.8×10^5^ cells) were cultured with or without heat-killed b240 (4.7×10^6^ counts), Pam3CSK4 (1 μg/ml), LPS (1 μg/ml), or ODN 1826 (1 μg/ml) for 4 days. IL-6 concentrations in the culture supernatants were determined by cytometric bead array. Data are expressed as mean ± SEM (n = 3). (TIF) [file pone.0091857.s003.tif]

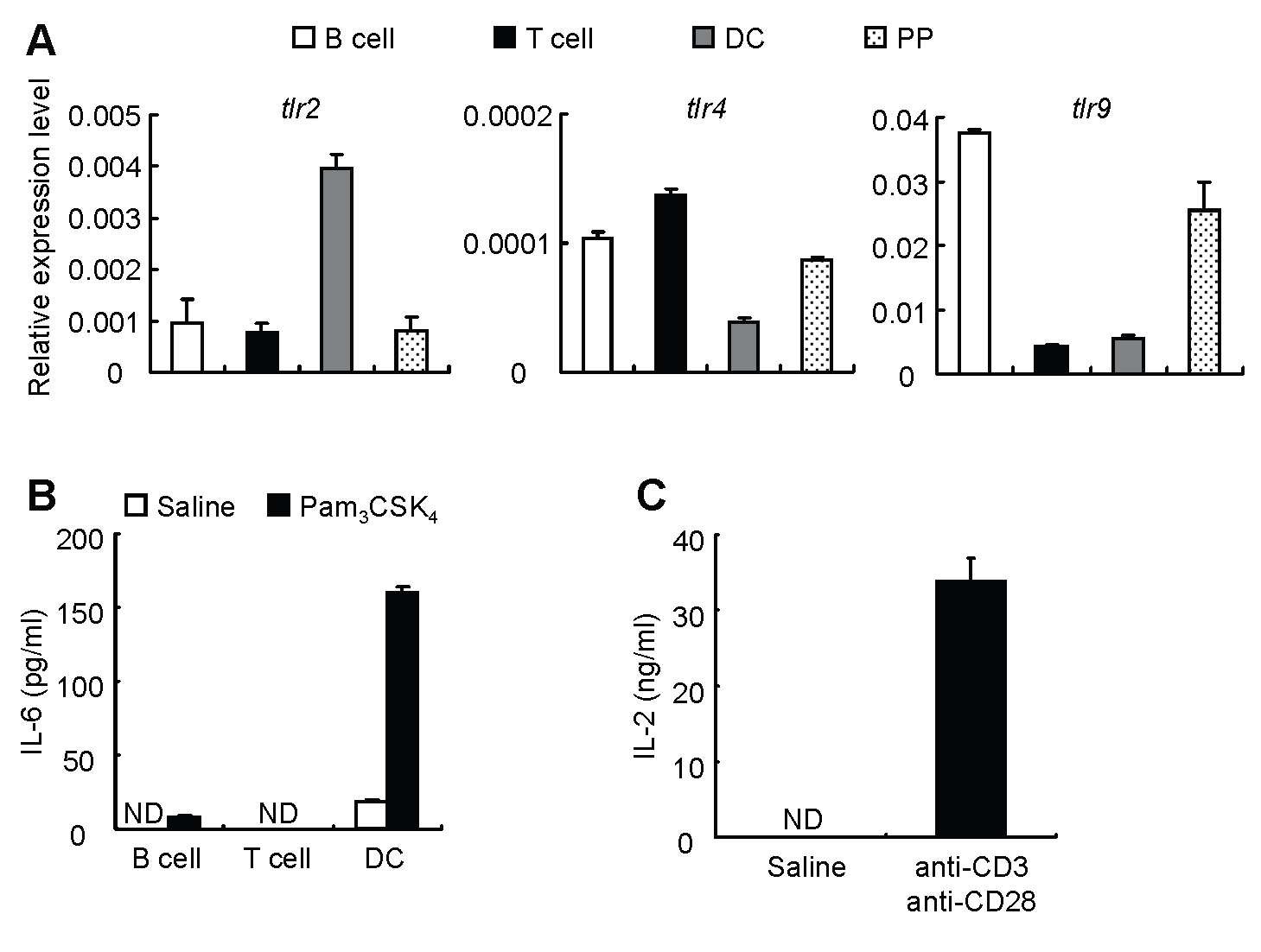

Supplement: Figure S2 — TLR expression and function in CD11c+B220− DCs, CD4+ T cells, and CD19+ B cells from PPs. (A) Purified CD11c+B220− DCs, CD4+ T cells, CD19+ B cells from PPs, and PP cells, were analyzed for gene expression levels of tlr2, 4, and 9. Expression was determined as fold induction compared with the β-actin housekeeping gene. Data are expressed as mean ± SD (n = 3). (B) Purified CD11c+B220− DCs, CD4+ T cells, or CD19+ B cells (1×10^5^ cells) from the PPs were cultured with or without Pam3CSK4 (1 μg/ml) in a 96-well flat-bottomed plate for 3 days and then IL-6 concentrations in the culture supernatants were determined by cytometric bead array (CBA). (C) Purified CD4+ T cells (1×10^5^ cells) from the PPs were cultured with or without pre-coated anti-CD3 antibody and anti-CD28 antibody (1 μg/ml) in a 96-well round-bottomed plate for 3 days, and then IL-2 concentrations in the culture supernatants were determined by CBA. (B, C) Data are expressed as mean ± SEM (n = 3). (TIF) [file pone.0091857.s004.tif]

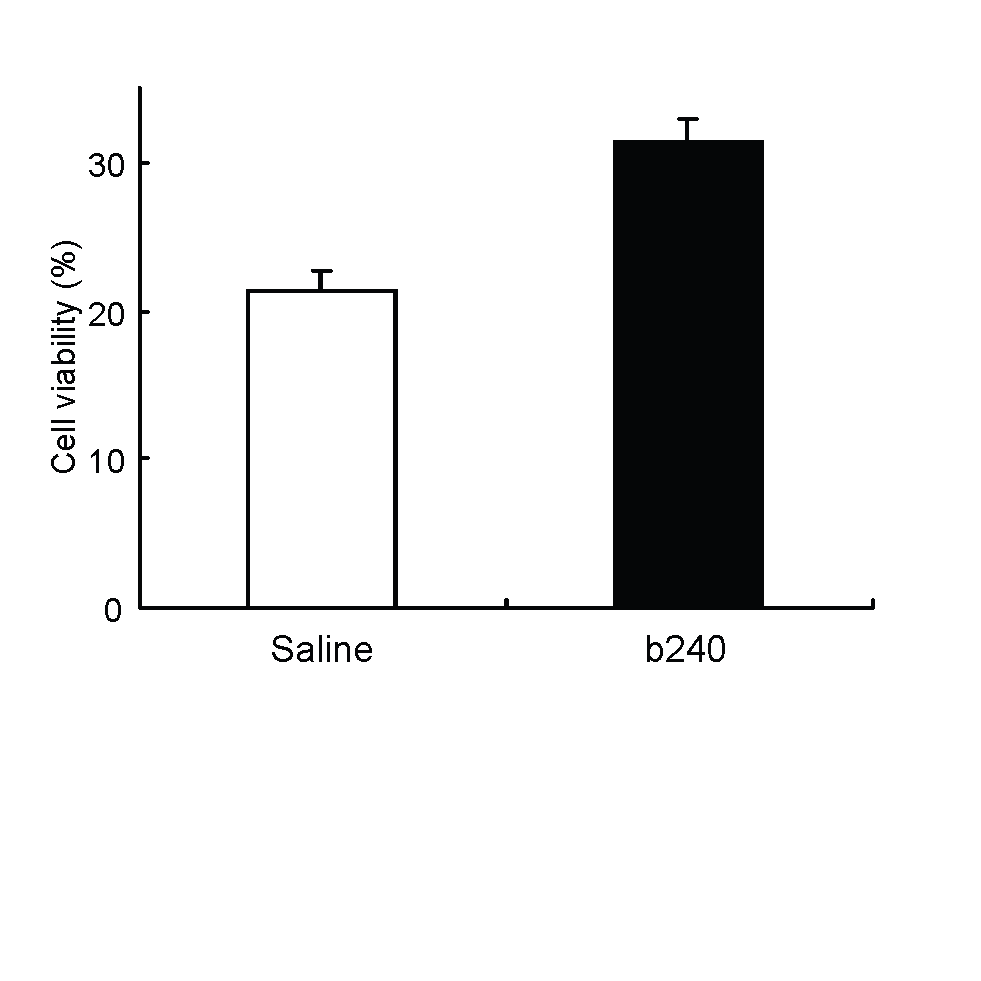

Supplement: Figure S3 — The effect of b240 on the PP cell viability. PP cells (5.8×10^5^ cells) were cultured with or without heat-killed b240 (4.7×10^6^ counts) for 4 days and then cell viability was evaluated by the Trypan blue dye exclusion test. Data are expressed as mean ± SEM (n = 5). (TIF) [file pone.0091857.s005.tif]

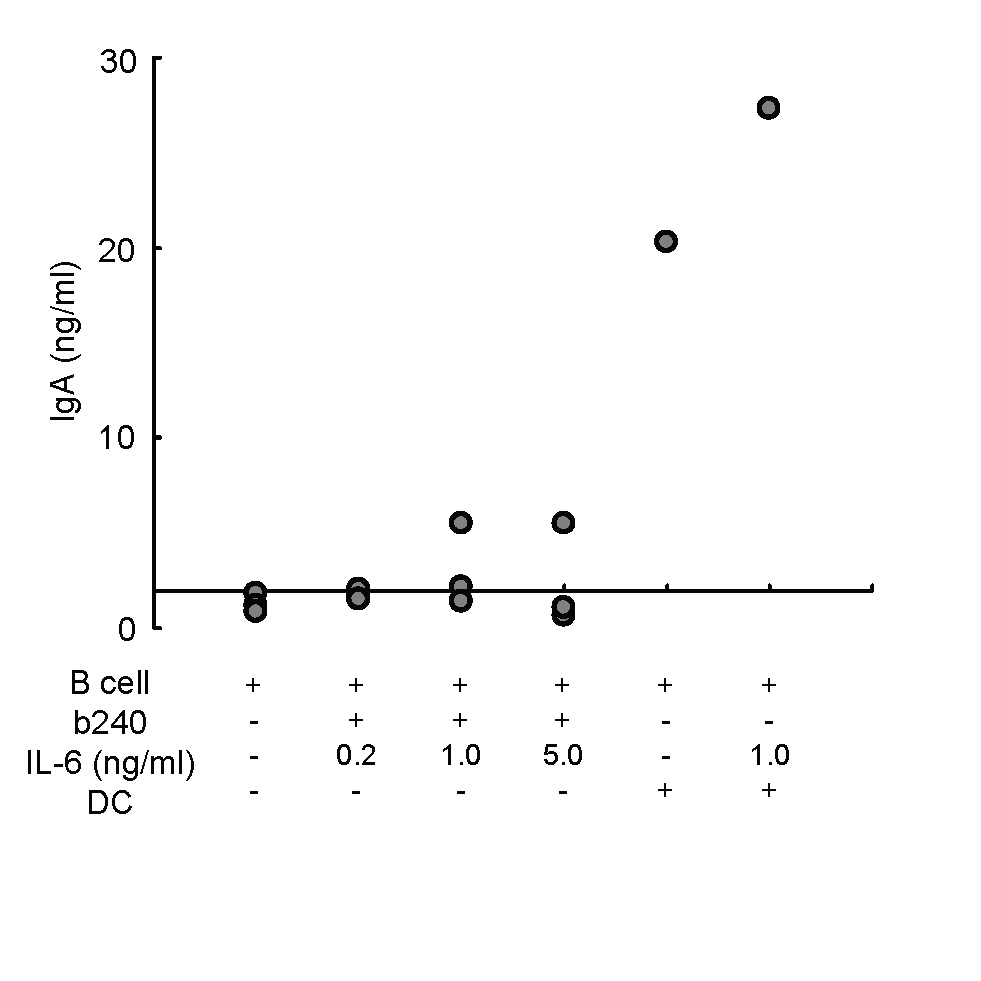

Supplement: Figure S4 — IgA production from b240- and IL-6-treated PP B cells. PP IgD+ B cells (2×10^5^ cells) were cultured with or without PP CD11c+B220− DCs (5×10^4^ cells), heat-killed b240 (1.6×10^6^ counts), or rIL-6 (0.2, 1.0, 5.0 ng/ml) for 7 days, and then IgA concentrations in the supernatants were determined by ELISA. The x-axis indicates the detection limit for IgA. (TIF) [file pone.0091857.s006.tif]
